# The role of alternative oxidase in the maintenance of cellular redox balance under hypoxia via participation in nitric oxide turnover

**DOI:** 10.1093/jxb/eraf021

**Published:** 2025-01-22

**Authors:** Abir U Igamberdiev, Natalia V Bykova

**Affiliations:** Department of Biology, Memorial University of Newfoundland, St. John’s, NL, A1B 3X9, Canada; Morden Research and Development Centre, Agriculture and Agri-Food Canada, Morden, MB, R6M 1Y5, Canada; INRA, France

**Keywords:** Alternative oxidase, hypoxia, mitochondria, nitric oxide, phytoglobin, redox regulation, seed germination

## Abstract

Alternative oxidase (AOX) regulates the level of reactive oxygen species and nitric oxide (NO) in plants. While it alleviates NO formation under normoxic conditions, there are several indications that in conditions of low oxygen, such as during seed germination before radicle protrusion, in meristematic stem cells, and in flooded roots, AOX can be involved in the production of NO from nitrite. Whereas the first reports considered this role as indirect, more evidence has since accumulated that AOX can act as a nitrite:NO reductase. Such activity of the structurally similar di-iron proteins in bacteria has been demonstrated. We review the literature on this topic and show that AOX can be induced under hypoxic conditions and participate in NO turnover via the phytoglobin–NO cycle. This results in the facilitation of glycolytic reactions by reoxidation of the glycolytically formed NADH and diverting the glycolytic carbon toward the formation of alanine and other amino acids. Pyruvate formed in glycolysis can activate AOX and facilitate its operation under these conditions. It is concluded that AOX is an important player in the hypoxic response in plants that regulates the redox level by participating in NO turnover as a nitrite:NO reductase in cooperation with nitrate reductase and phytoglobin.

## Teaser

Alternative oxidase is essential under oxygen-limiting conditions for regulating cellular redox balance via the participation in nitric oxide (NO) turnover as a nitrite:NO reductase in cooperation with nitrate reductase and phytoglobin.

## Introduction

Alternative oxidase (AOX) is a di-iron protein catalyzing the redox reaction of oxygen reduction to water not coupled to the generation of a proton gradient (Siedow *et al.*, 1995). It was identified as a quinol oxidase by Rich (1978) and isolated and characterized from plant mitochondria by Elthon and McIntosh (1987). AOX is encoded in the nuclear genome by the family of *Aox* genes having two subfamilies *Aox1* and *Aox2* in dicots and only one subfamily *Aox1* in monocots (Considine *et al.*, 2002). The first subfamily is mostly involved in the adaptation to stress, while the function of the second family is related to plant development (Kumari *et al.*, 2019). The use of alternative electron acceptors by AOX (such as nitrite) is not as well explored for AOX as for other metal-containing proteins such as hemeproteins, although there are several indications that AOX is capable of nitric oxide (NO) production from nitrite (Vishwakarma *et al.*, 2018; Jayawardhane *et al.*, 2020; Kumari *et al.*, 2022).

Mitochondria of higher plants and algae were shown to catalyze the reaction of nitrite reduction to NO (Igamberdiev *et al.*, 2014, and references therein). This reaction was demonstrated for cytochrome *c* oxidase (COX; Castello *et al.*, 2006, 2008), cytochrome *c* (Basu *et al.*, 2008), and Complex III (Kozlov *et al.*, 1999; Alber *et al.*, 2017). The latter can potentially perform the reaction from both sides of the inner mitochondrial membrane (reviewed by Gupta *et al.*, 2018). In plants, in particular in root tissues, mitochondria represent the main site of NO production from nitrite under conditions of oxygen depletion (Gupta *et al.*, 2005; Gupta and Igamberdiev, 2011), and this may be related to AOX involvement in NO turnover (Jayawardhane *et al.*, 2020). In animal tissues, mitochondria also represent a significant source of NO formation from nitrite (Feelisch *et al.*, 2008). Moreover, mitochondria are capable of performing oxidative pathways of NO formation (reviewed in Igamberdiev *et al.*, 2014).

In the previous review paper on AOX (Kumari *et al.*, 2019), its role in the regulation of NO levels under normoxic and hypoxic conditions in plants was discussed. The current review deals with the structural aspects of the AOX molecule, which make possible the catalysis of NO production from nitrite, with the coordinated operation of AOX, nitrate reductase, and phytoglobin (Pgb); and with the role of AOX in anaerobic metabolism during seed germination. We conclude that AOX is important for the adaptation to hypoxic conditions in plants, which is achieved through the ability of AOX to regulate cellular redox levels via participation in NO turnover. This results in the increase in energy efficiency of cells under conditions of oxygen deprivation, which occur during various abiotic stresses and at different stages of plant development.

## Alternative oxidase: protein structure and the mechanism of redox catalysis

The class of di-iron proteins to which AOX belongs (Moore *et al.*, 1995) includes the hydroxylase subunit of methane monooxygenase, the R_2_ subunit of ribonucleotide reductase, ferritins, stearoyl-acyl carrier protein Δ^9^-desaturase, and rubrerythrin (Andersson and Nordlund, 1999). The active site of these proteins consists of a binuclear Fe center coordinated by two histidines and four carboxylate residues of Glu or Asp (two copies of the primary sequence motif D/E-X-X-H). Three conserved EXXH motifs were previously detected in the AOX protein sequence (Andersson and Nordlund, 1999). The crystal structure of *Trypanosoma brucei* AOX revealed the non-heme di-iron carboxylate active site, which was buried within a four-helix bundle and ligated by four Glu residues, and in proximity to the location of a highly conserved Tyr residue critical for catalytic activity (Shiba *et al.*, 2013). Two ubiquinol molecules bind consecutively in the same pocket containing Arg residues, whereas two hydrophobic cavities present in each AOX monomer participate in AOX catalysis, in which the formation of high-valent ferryl/ferric species and the tyrosyl radical drive quinol oxidation via electric field effects (Shiba *et al.*, 2019; Saura *et al.*, 2024).

The di-iron center contains two iron atoms connected either by a µ-oxo- or µ-hydroxo-bridging ligand. In addition to this ligand, at least one other bridging ligand is a carboxylate group, with the remaining iron ligands being N atoms from His residues and O atoms from Glu or Asp residues. Both of the His residues and one of the two carboxylates serve as monodentate ligands to the iron atoms in the binuclear cluster, while the second carboxylate acts as a bidentate ligand, bridging the two iron atoms. The active site of AOX is located deep inside the hydrophobic cavity (Moore *et al.*, 1995; Andersson and Nordlund, 1999; Shiba *et al.*, 2013). The proposed AOX active site structure was initially suggested by Moore *et al.* (1995), further developed by Andersson and Nordlund (1999), and advanced in detail after the crystal structure of AOX was established (Shiba *et al.*, 2013, 2019). The conserved Tyr residue is aligned with the hydrophobic pocket formed by Ile and/or Phe and plays a central role in electron transfer from ubiquinol by forming the long-lived Tyr radical in the course of the reaction (Berthold *et al.*, 2000). There are two bridging groups, an oxo- and a Glu residue between the two iron atoms. The two sets of di-iron sites in the AOX dimer act independently in catalyzing ubiquinol oxidation and transfer of electrons (Berthold *et al.*, 2000; Moore *et al.*, 2013; Young *et al.*, 2014, 2020).

The reaction of nitrite reduction to NO can readily occur in the presence of reductants and, at low pH, it takes place even non-enzymatically in the presence of ascorbate or other reducing molecules such as phenolic compounds, for example in the apoplastic space of germinating seeds (Bethke *et al.*, 2004). The reaction is significantly facilitated via levering coordination of NO_2_^−^ to Fe centers, which lowers the reduction potential of NO_2_^−^, making electron transfer more favorable (Hosseininasab *et al.*, 2022). In this coordination, the nitrite radical dianion NO_2_^2−^ is formed, where N has an oxidation state of N(II). Protonation of NO_2_^2−^ leads to the release of NO.

Previously, the catalysis of NO formation from nitrite was discussed mostly in connection with reactions that involved hemeproteins and iron–sulfur clusters (Li *et al.*, 2004, 2008; Omar and Webb, 2014). Furthermore, molybdocofactors of nitrate reductase (Dean and Harper, 1986; Bender and Schwartz, 2018) and xanthine oxidase (Li *et al.*, 2008) were shown to be involved in NO production, however they operate in these enzymes together with iron-containing domains. In bacteria, nitrite reduction is associated with siroheme-containing enzymes (NirBD), cytochrome *c* hemoproteins (NrfA and NirS), and copper-containing enzymes (NirK) (Crack *et al.*, 2022). Only recently it was shown that NO generation from nitrite is associated with di-iron proteins such as YtfE (Crack *et al.*, 2022). Other functions of this protein may be related to the donation of iron for the repair of iron–sulfur clusters damaged by nitrosative stress, the release of NO from nitrosylated iron, and the reduction of NO to N_2_O. Although the rate of the latter reaction is very low, in the reaction of nitrite reduction to NO this protein possesses a high affinity for nitrite and a significant rate of NO formation (Crack *et al.*, 2022). It was also shown that the di-iron proteins in bacteria catalyze the reverse reaction of NO detoxification by forming nitrite at the di-iron center (Poptic *et al.*, 2023). Some di-iron proteins (such as Ytfe) primarily catalyze the formation of NO, while others (such as Mka-HPL) oxidize NO to nitrite (Poptic *et al.*, 2023).

Hence, as AOX has a structural similarity to methane monooxygenase (Moore *et al.*, 1995), it is worth considering its operation in the context of metabolism in methane-oxidizing bacteria. The nitrite-reducing anaerobic methanotrophs *Methylomirabilis* contain the genes encoding cyanide-insensitive AOX (Wu *et al.*, 2011). Nitrite-dependent anaerobic methane oxidation by these microorganisms is linked to the production of oxygen intracellularly by the disproportionation of two NO molecules into N_2_ and O_2_. The produced O_2_ is then used in the aerobic methane oxidation pathway and during respiration (Shen *et al.*, 2015). The divergence from methane monooxygenase to AOX could take place already in this group of bacteria, and the simultaneous operation of methane oxidation and terminal oxidases provides the bioenergetic adaptation for a more efficient energy transfer within the metabolic pathways of these microorganisms.

When AOX reacts with oxygen, the metal center of the reduced di-ferrous form of the enzyme reacts with O_2_, forming a di-ferric-peroxide intermediate. The next step is the formation of the oxo-ferryl and then hydroxo-ferryl species, with the final release of the water molecule (Andersson and Nordlund, 1999). The two sets of di-iron sites in the AOX dimer operate independently of each other to generate the functional oxygen-reducing species (Moore *et al.*, 1995). In the case of nitrite binding to Fe centers, the radicals of the nitrite dianion NO_2_^2−^ could be generated instead of the formation of a di-ferric-peroxide intermediate. Further protonation of the nitrosylated Fe centers via the transfer of electrons and protons from the ubiquinol-binding sites results in NO release. This mechanism requires further clarification, but it should have a similarity to the enzymatic process established for the di-iron protein Ytfe in *Escherichia coli* (Crack *et al.*, 2022). Similarly to the Ytfe protein, the di-iron site of AOX can exist in a mixture of forms, including nitrosylated and nitrite bound. The addition of nitrite to di-ferrous YtfE resulted in nitrosylated YtfE and the release of NO. The Lewis acidity of the Fe center facilitates the transfer of electrons and protons to nitrite (Hosseininasab *et al.*, 2022).

In this reaction, the action of tyrosine-OH in coordination with histidine and one of the carboxylic amino acids can provide an efficient reduction mechanism of AOX. We can suppose, that similarly to the AOX reaction of reducing oxygen (Young *et al.*, 2020; Yamasaki *et al.*, 2021), the reaction of nitrite reduction to NO is facilitated by pyruvate. Other oxo-acids can also be involved in the activation of AOX (Selinski *et al.*, 2018). Pyruvate can originate from glycolysis, which is activated under the conditions of hypoxia, where nitrite replaces oxygen as an acceptor of electrons (Igamberdiev and Hill, 2004, 2009; Igamberdiev *et al.*, 2010).

## Alternative oxidase and nitrite reduction by plant mitochondria

The role of AOX in the adaptation to the conditions characterized by NO formation was established when it was found that in contrast to the cytochrome pathway, AOX is not inhibited by NO (Millar and Day, 1996). However, this fact cannot help much in sustaining respiration under hypoxia because the affinity of AOX for oxygen as compared with COX is 1–2 orders of magnitude lower (Millar *et al.*, 1994; Affourtit *et al.*, 2001). On the other hand, it was demonstrated that AOX is important for the adaptation and survival under low oxygen conditions (Zabalza *et al.*, 2009; reviewed in Kumari *et al.*, 2019; Popov *et al.*, 2021). One explanation suggested by Zabalza *et al.* (2009) is based on the observation that the cytochrome *c* pathway exhibits a non-linear inhibition upon a decline in oxygen level, which results in progressively faster decrease in the respiratory rate upon decreasing oxygen availability, while the inhibition of the AOX pathway shows only the linear component of the adaptive response. However, this may not be sufficient to explain the importance of AOX in plant adaptation to low oxygen.

In the studies with *Chlamydomonas* (Tischner *et al.*, 2004) and then with tobacco cell suspension cultures (Planchet *et al.*, 2005), it was shown that the inhibitors of the mitochondrial electron transport chain (ETC) suppress NO production from nitrite. It was suggested that mitochondria represent the main site of nitrite reduction to NO (Planchet *et al.*, 2005; Blokhina and Fagerstedt, 2010), and that COX and AOX both contribute approximately equally to NO formation (Gupta and Igamberdiev, 2011; Igamberdiev *et al.*, 2014). This conclusion was derived from the observation that both cyanide, the inhibitor of COX, and salicylhydroxamic acid (SHAM), the inhibitor of AOX, are effective in suppressing NO formation from nitrite. It was suggested, however, that since SHAM is not a very specific inhibitor and can suppress peroxidases and other redox proteins (Rich *et al.*, 1978), the effect of SHAM might not be directly related to the inhibition of AOX. Nevertheless, the use of transgenic lines differentially expressing AOX and the detection of NO generation by chemiluminescence and DAF-FM (4-amino-5-methylamino-2’,7’-difluorofluorescein) fluorescence provide additional evidence for the contribution of AOX to NO production from nitrite. Table 1 presents information on possible AOX involvement in the reduction of nitrite to NO, which is based on the original experimental studies using transgenic tobacco plants with differential expression of AOX.

**Table 1.** Studies providing evidence for the participation of AOX in nitric oxide production

Effect	Method of detection	Object	Reference
~50% inhibition of NO production from nitrite by SHAM	Chemiluminescence detection	*Chlorella sorokiniana* (nitrite reductase mutant)	Tischner *et al.* (2004)
Inhibition of NO production from nitrite by SHAM	Chemiluminescence detection	Leaves and isolated mitochondria from cell suspension cultures of *Nicotiana tabacum*	Planchet *et al.* (2005)
Inhibition of NO production from nitrite by SHAM in root mitochondria	Chemiluminescence detection	Isolated root and leaf mitochondria of *Arabidopsis thaliana*, *Hordeum vulgare*, *Pisum sativum*, *Nicotiana tabacum*	Gupta *et al.* (2005)
AOX overexpression lines generated more NO than AOX antisense lines, SHAM inhibition	DAF-FM fluorescence	AOX transgenic lines of *Arabidopsis thaliana*	Vishwakarma *et al.* (2018)
AOX overexpression lines generated more NO than AOX antisense lines, SHAM inhibition	Chemiluminescence detection of NO, detection of *S*-nitrosated proteins and glutathione, NO-dependent inhibition of aconitase	AOX transgenic lines of *Nicotiana tabacum*	Jayawardhane *et al.* (2020)
AOX overexpression lines generated more NO from nitrite than AOX antisense lines, SHAM inhibition	DAF-FM fluorescence, chemiluminescence detection of NO	Submerged seedlings and anaerobically germinated seeds of deepwater *Oryza sativa* incubated with nitrite	Kumari *et al.* (2022)
NO generation in AOX overexpression lines, and the induction of GABA shunt and phosphorylated serine pathway	Chemiluminescence detection of NO	AOX transgenic lines of *Nicotiana tabacum*	Zafari *et al.* (2022a)
AOX overexpression lines generated more NO than AOX antisense lines	Chemiluminescence detection of NO, chemical detection of *S*-nitrosated proteins	AOX transgenic lines of *Nicotiana tabacum*	Zafari *et al.* (2022b)

**Fig. 1.**
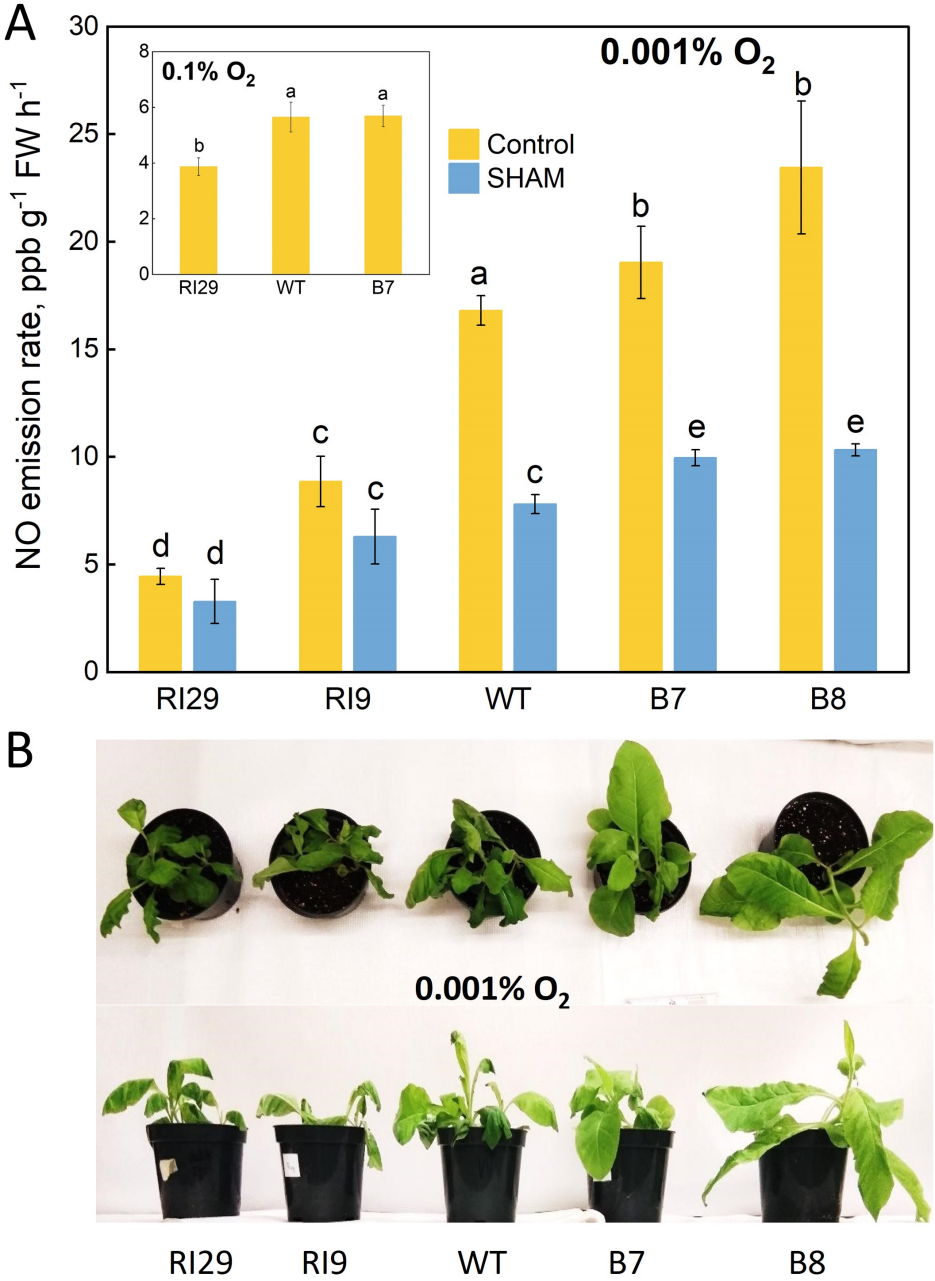
The rates of NO production by tobacco (*Nicotiana tabacum* L.) plants differentially expressing AOX. NO emissions from the detached leaves incubated in nitrate solution (A) in nitrogen gas with 0.001% and 0.1% oxygen (inserted graph); and the images of transgenic plants after a 4 h incubation in nitrogen gas with 0.001% oxygen (B) are shown. Among the transgenic lines used in this study, RI29 is the most effective knockdown (no detectable leaf AOX protein), while faint amounts of leaf AOX protein can be detected in RI9. The lines B8 and B7 are both effective AOX overexpressors, with B8 showing slightly higher amounts of AOX protein than B7. The conditions of the experiment were described in Jayawardhane *et al.* (2020). Image (B) is reproduced from this paper, graph (A) is based on the set of data of this paper (*n*=5). Data in the insert in A is from Cochrane (2020).

Tobacco plants overexpressing AOX featured a higher capacity for NO scavenging under normoxia (Cvetkovska and Vanlerberghe, 2012; Alber *et al.*, 2017), and produced more NO from nitrite under almost anoxic conditions (nitrogen atmosphere with ~0.001% O_2_) as compared with AOX knockout plants (Jayawardhane *et al.*, 2020). The same effect was seen under hypoxic conditions (0.1% O_2_) (Cochrane, 2020), while SHAM application led to the decrease in NO production to levels close to those observed in the knockout plants (Fig. 1A). The plants overexpressing AOX were more viable after 4 h incubation in a nitrogen atmosphere (Fig. 1B). In a previous study, Vishwakarma *et al.* (2018) demonstrated in Arabidopsis the same effect of AOX overexpression on NO production from nitrite as was observed in transgenic tobacco plants. Evidently, these observations can be considered as a strong indication of a direct participation of AOX in the generation of NO from nitrite. The recent discovery of the catalysis of this reaction by the Ytfe protein from *E. coli* (Crack *et al.*, 2022), an enzyme that is structurally different from plant mitochondrial AOX but belongs to the same group of di-iron proteins, makes this possibility very plausible. Structural and physico-chemical studies of nitrite binding and conversion by AOX from the plant mitochondrial ETC are yet to be accomplished in order to reveal the molecular mechanisms of this reaction.

The information obtained to date in plants demonstrates that in contrast to its function under normoxia, AOX has a specific role under hypoxia, where AOX can facilitate nitrite-dependent NO production. Via participation in this reaction, AOX can contribute to the Pgb–NO cycle by driving it through NO production. As a result, despite the fact that AOX is not scavenging but producing NO, it contributes to the increase in energy efficiency under hypoxia by lowering the redox level of hypoxic plant cells. In all reactions of the Pgb–NO cycle, NAD(P)H is oxidized. These reactions include nitrate reductase, nitrite:NO reduction (operation of AOX or COX in concert with the Complex I or alternative dehydrogenases), and metphytoglobin reductase, whose exact nature has yet to be established in plants (Igamberdiev *et al*., 2006; Sainz *et al.*, 2013; Jokipii-Lukkariet *et al*., 2016). While a major part of NADH in hypoxic cells is generated via glycolysis, the oxidation of glycolytic NADH in the Pgb–NO cycle would result in bypassing the lactic and ethanolic fermentation, and the diversion of glycolytic carbon towards the formation of Ala and other amino acids.

## Operation of glycolytic fermentation and the role of alternative oxidase in the diversion of glycolytic carbon to amino acids

The Pgb–NO cycle and glycolytic fermentation can operate in a cooperative manner. When the glycolytic NADH is oxidized in the nitrate reductase, nitrite:NO reductase, and metphytoglobin reductase reactions of the cycle, the pool of pyruvate produced in glycolysis can be further converted into alanine, which is one of the most accumulated compounds under hypoxic conditions (Fan *et al.*, 1988). Muench and Good (1994) demonstrated that Ala accumulation is caused by the hypoxic induction of alanine aminotransferase. Moreover, Pandey *et al.* (2019) observed an increased incorporation of ^13^C into soluble Ala in germinating seeds of chickpeas upon treatment with an NO donor. These studies provide evidence that the carbon and nitrogen metabolic pathways in the bioenergetic system of hypoxic cells cooperate towards the support of energetic needs under oxygen deficiency, in particular in germinated seeds. This cooperation was even more pronounced in hypoxia-tolerant plants (Jayawardhane *et al.*, 2020).

Glutamate dehydrogenase (GDH) isoforms that preferably operate in the directions of ammonia assimilation act as anti-stress enzymes in ammonia detoxification and Glu production for Pro synthesis (Skopelitis *et al.*, 2006) and γ-aminobutyric acid (GABA) synthesis (Eprintsev *et al.*, 2024). The predominance of α-subunits in the GDH structure results in a higher affinity for Glu, while the presence of β-subunits increases the affinity for 2-oxoglutarate (Purnell *et al.*, 2005; Purnell and Botella, 2007; Tercé-Laforgue, 2013). In lupine roots, which experience hypoxia more than other tissues, the β-GDH subunit was the only GDH polypeptide present (Lehmann *et al.*, 2011). The role of GDH in ammonium assimilation was established, in particular, in submerged plants experiencing oxygen deprivation (Xian *et al.*, 2020). Thus, Glu necessary for the amination of glycolytic pyruvate can be provided by the action of GDH (Skopelitis *et al.*, 2006; Eprintsev *et al.*, 2024).

The reciprocal regulation of the Pgb–NO cycle and glycolysis by gibberellin and abscisic acid, respectively, under hypoxic conditions has been demonstrated (Nie *et al.*, 2022), indicating that both processes are metabolically linked. The reoxidation of the glycolytic NADH in the Pgb–NO cycle can lead to AOX activation by the glycolytic pyruvate, which can be accumulated before its amination to Ala. On the other hand, NO production under hypoxia inhibits aconitase, which converts citrate to isocitrate, so citrate accumulates and induces AOX transcription. This leads to the stimulation of hypoxic metabolism and accumulation of amino acids (Gupta *et al.*, 2012). The indirect induction of AOX by NO was shown in several organisms (Ederli *et al.*, 2006; Fu *et al.*, 2010; Royo *et al.*, 2015), and the inhibition of aconitase represents an important mechanism for hypoxia-induced maintenance of partial electron flow through the mitochondrial ETC and scavenging of NO by AOX.

The alternative pathway of glycolysis, the phosphorylated pathway of serine biosynthesis (PPSB), is associated with plastids, leads to the formation of the amino acids Ser and Gly, and is also linked to the GABA pathway (Igamberdiev and Kleczkowski, 2018). The rate of NO emission in tobacco plants under hypoxia was correlated with AOX content and with the accumulation of amino acids (Zafari *et al.*, 2022a). This amino acid accumulation was associated with the increased expression of the enzymes of the PPSB metabolic process. The induction of this process occurred in the first hours of hypoxia, while the induction of the GABA pathway followed several hours later. This study established that high rates of NO turnover were accompanied by rapid induction of genes involved in the PPSB pathway, which is important for the maintenance of carbon, nitrogen, and energy metabolism under hypoxia. It was also shown that AOX controls the interaction of NO, reactive oxygen species, and ethylene under hypoxia, triggering a coordinated downstream defensive response in the adaptation to low oxygen (Zafari *et al.*, 2022b). The hypoxically induced ethylene biosynthesis genes encoding 1-aminocyclopropane-1-carboxylic acid (ACC) synthase, ACC oxidase, and ethylene-responsive factors (ERFs) correlated with AOX and NO levels and was shown to regulate the flux of glycolytic fermentation.

**Fig. 2.**
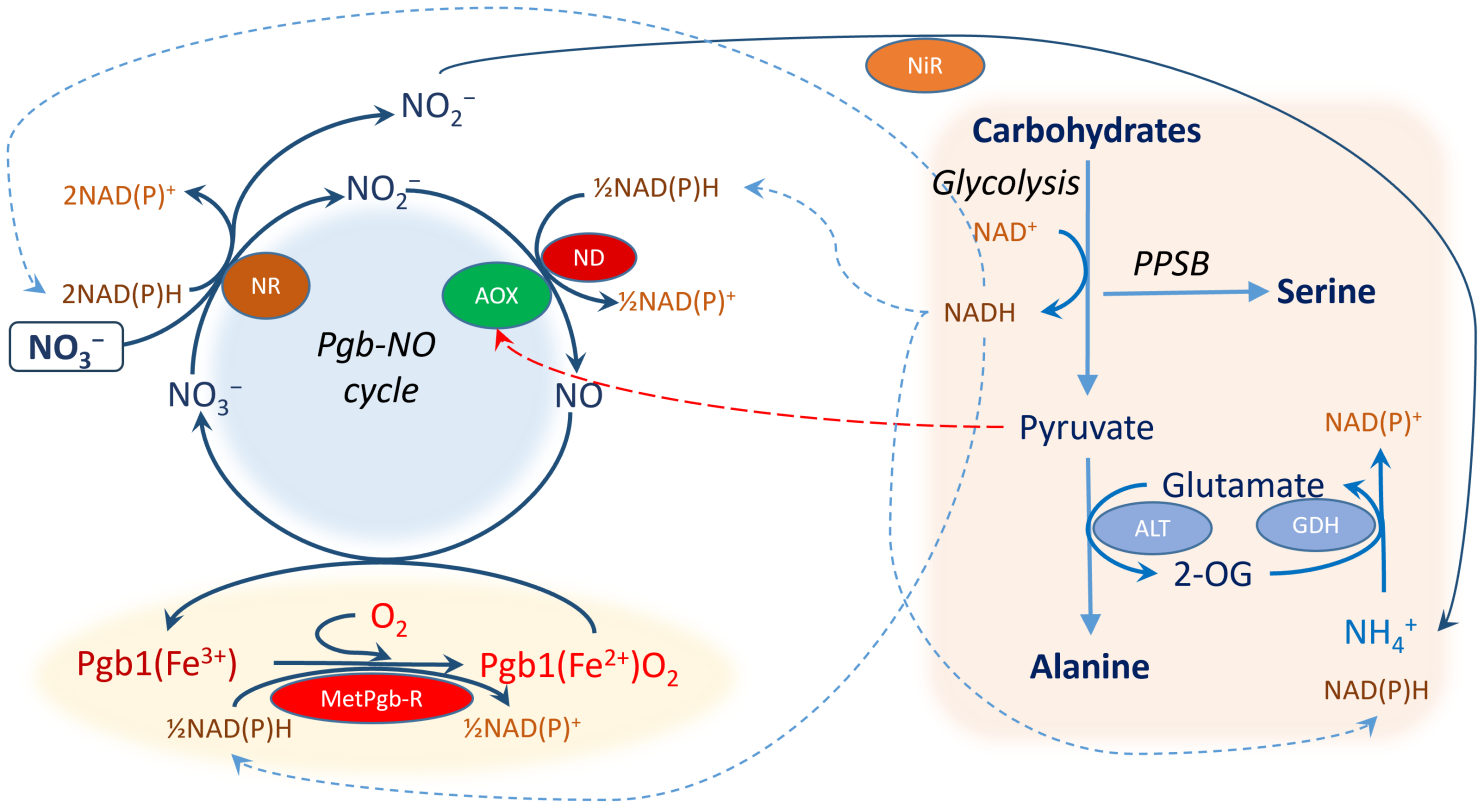
Participation of AOX in the Pgb–NO cycle as a part of the nitrite:NO reductase system of the mitochondrial electron transport chain and the role of the cycle in the reoxidation of glycolytic NADH. Abbreviations: ALT, alanine aminotransferase; GDH, glutamate dehydrogenase; MetPgb-R, metphytoglobin reductase; ND, NAD(P)H dehydrogenase (Complex I or the alternative dehydrogenases); NR, nitrate reductase; NiR, nitrite reductase; PPSB, the phosphorylated pathway of serine biosynthesis.

The participation of AOX in the Pgb–NO cycle as a part of the nitrite:NO reductase system of the mitochondrial ETC and the role of this cycle in the reoxidation of glycolytic NADH are shown in Fig. 2. AOX can function in coordination with either NADH oxidation via Complex I or alternative NAD(P)H dehydrogenases. The operation of glycolysis leads to Ala accumulation via its main pathway or to serine accumulation via PPSB. The pathways depicted in Fig. 2 are supported by the consumption of nitrate, which can be reduced to ammonium ion via classical nitrite reductase or to NO via the nitrite:NO reductase in the mitochondrial ETC. Thus, the role of AOX under hypoxic conditions is associated with the redox level control through its participation in the Pgb–NO cycle and with the metabolic shift to amino acid synthesis. This directs the flow of glycolytic carbon to the biosynthetic processes and results in the avoidance of negative consequences of lactic or ethanolic fermentation such as acidification of the cell in the first case and the loss of carbon in the second.

## Alternative oxidase induction under hypoxic conditions

There are several indications that AOX protein is induced not only in response to a high redox level during oxygen-rich conditions but also under hypoxia. Seed germination at the stage from seed imbibition to radicle protrusion represents the hypoxic phase of plant development. It is characterized by a fast consumption of internal oxygen that leads to a limitation of respiratory capacity and thus to the suppression of mitochondrial respiration and a high cellular redox potential (Bykova and Igamberdiev, 2024). The operation of mitochondria in the absence of oxygen using nitrite as a terminal electron acceptor is a possibility under these conditions, which can work for balancing the redox level and meeting energy demands (Igamberdiev and Hill, 2004; Samant *et al*., 2024).

A few species of higher plants such as rice (*Oryza* sp.), barnyard grass (*Echinochloa crus-galli*), and the African legume *Erythrina caffra* are even able to germinate and grow under strict anoxia (Kennedy *et al.*, 1992). Despite oxygen limitation during germination, it was shown that AOX could be important and strongly induced at the germination stage. This was shown in germinating chickpea (*Cicer arietinum*) (Pandey *et al.*, 2019; Joseph *et al.*, 2024). In germinating pea (*Pisum sativum*) seeds, the inhibition of AOX had a suppressive effect on germination, and two of three AOX isoforms (PsAOX1 and PsAOX2d) were shown to be induced in the first hours of germination (Rodrigues *et al.*, 2023). Anaerobic germination of rice was demonstrated to be strongly dependent on AOX expression. AOX inhibition by SHAM retarded the anoxic growth of rice mediated by nitrite. It was suggested that the NO turnover via the Pgb–NO cycle represents an important mechanism for bioenergetics of anaerobically germinating rice seeds, and its role also remains important after germination as nitrite improves the submergence tolerance of seedlings (Kumari *et al.*, 2022). It was also shown that the isoform AOX1a in rice is critical for seed viability during seed storage (Ji *et al.*, 2023).

Accelerated promotion of seed germination in Arabidopsis facilitated by the application of H_2_S was associated with the induction of the AOX pathway (Fang *et al.*, 2022). AOX2 mRNA accumulated already in dry seeds of Arabidopsis, and the gene for AOX2, which consists of five exons unlike other AOX genes, was transcribed at an early stage during germination (Saish *et al.*, 2001). It was recently demonstrated that AOX supports fermentation during the first 12 h after imbibition, and its effect is highly expressed in coordination with the exogenous sucrose application to seeds of different plant species (Bharadwaj *et al.*, 2021). Arabidopsis mutants deficient in Complex IV keep the viability of isolated embryos via the induction of AOX and alternative rotenone-insensitive NAD(P) dehydrogenases (Dahan *et al.*, 2014).

In cowpea (*Vigna unguiculata*), the co-expression of *Aox1* and *Aox2b* genes encoding two AOX isoforms takes place during the early phase of seed germination and in response to abiotic stresses. The dual nature of *Aox2b* expression (constitutive and induced) was revealed, which suggests that the constitutive *Aox2b* gene of *V. unguiculata* acquires inducible regulatory elements (Costa *et al.*, 2010). In carrot seeds (Mohanapriya *et al.*, 2019), it was shown that AOX is important for developmental plasticity and yield stability. Considering the low affinity of AOX for oxygen, its role during seed germination is, at least in part, related to AOX participation in NO turnover via the Pgb–NO cycle, which was firmly established in the case of anaerobically germinating rice (Kumari *et al.*, 2022).

**Fig. 3.**
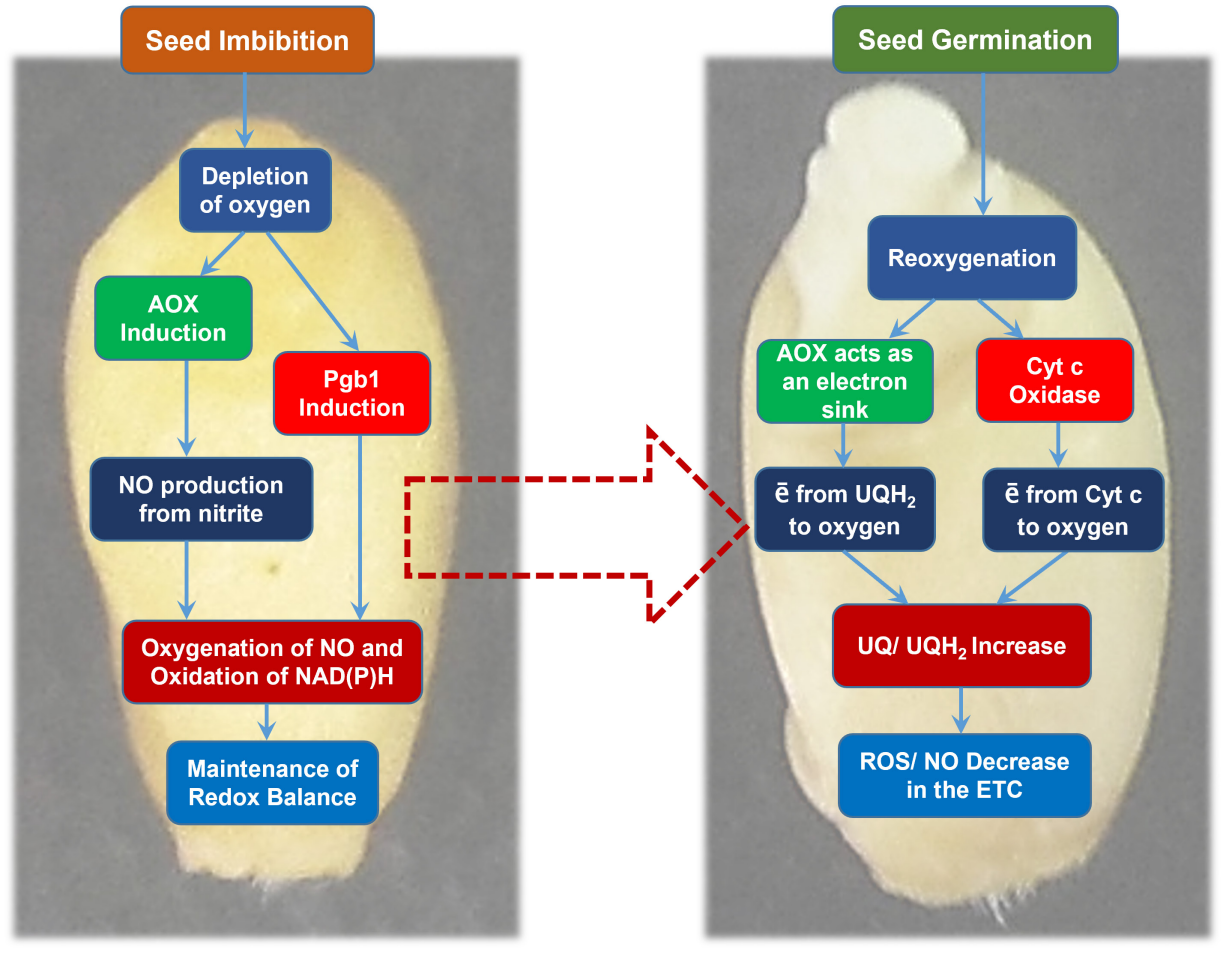
Sequence of events during seed germination related to AOX and Pgb induction and leading to the maintenance of redox balance.

The sequence of events during seed germination that is related to AOX induction and its participation in the redox regulation of metabolism is shown in Fig. 3. The coordinated expression of AOX and class 1 phytoglobin (Pgb1) leads to the promotion of NO turnover and continuous oxidation of NAD(P)H in the Pgb–NO cycle. AOX can be an important player in the process of seed germination at the stage of oxygen depletion before radicle protrusion. After the seed coat rupture in the process of seedling development, the role of AOX changes to the essential respiratory function of the electrons passing through the ETC directly to molecular oxygen, which decreases the reduction level of ubiquinone and prevents the formation of reactive oxygen species and NO.

Besides germinating seeds, another important site of oxygen depletion is represented by the compact rapidly dividing meristematic cells (Mira *et al.*, 2020). These cells can actively produce NO, and scavenge it by Pgb, which results in the maintenance of meristem redox levels and avoidance of meristem cell death (Mira *et al.*, 2023). The specific localization of AOX was previously demonstrated in meristematic and xylematic tissues from developing soybean roots and hypocotyls (Hilal *et al.*, 1997). The authors suggested that AOX expression is linked to xylem differentiation. Further studies are needed to clarify the possible participation of AOX in NO turnover in meristematic cells and the maintenance of their functionality.

The hypoxic induction of AOX was reported in several organisms including parasitic ciliates (Folgueira *et al.*, 2020), fungi (Stanić *et al.*, 2013), and mollusks (Yusseppone *et al.*, 2018; Wang and Zhang, 2021). Sudden submergence of aerobically grown barley seedlings under water resulted in AOX suppression under flooding-induced hypoxic conditions in green leaves, although in roots the AOX capacity remained high (Skutnik and Rychter, 2009). On the other hand, the induction of particular AOX genes and their coordination with genes of other non-phosphorylating bypasses of the ETC were detected under abiotic stress conditions, reflecting the multiplicity of responses that affect alternative ETC components in plant cells (Clifton *et al.*, 2005). Wany *et al.* (2019) demonstrated that the induction of AOX under hypoxia in Arabidopsis is pronounced in the presence of nitrate, indicating its participation in nitrogen turnover. This led to the establishment of the role of AOX in anaerobic germination and growth of deepwater rice (Kumari *et al.*, 2022). Further studies will aim to demonstrate the ubiquitous AOX operation under hypoxic conditions via its involvement in nitrogen metabolism.

**Fig. 4.**
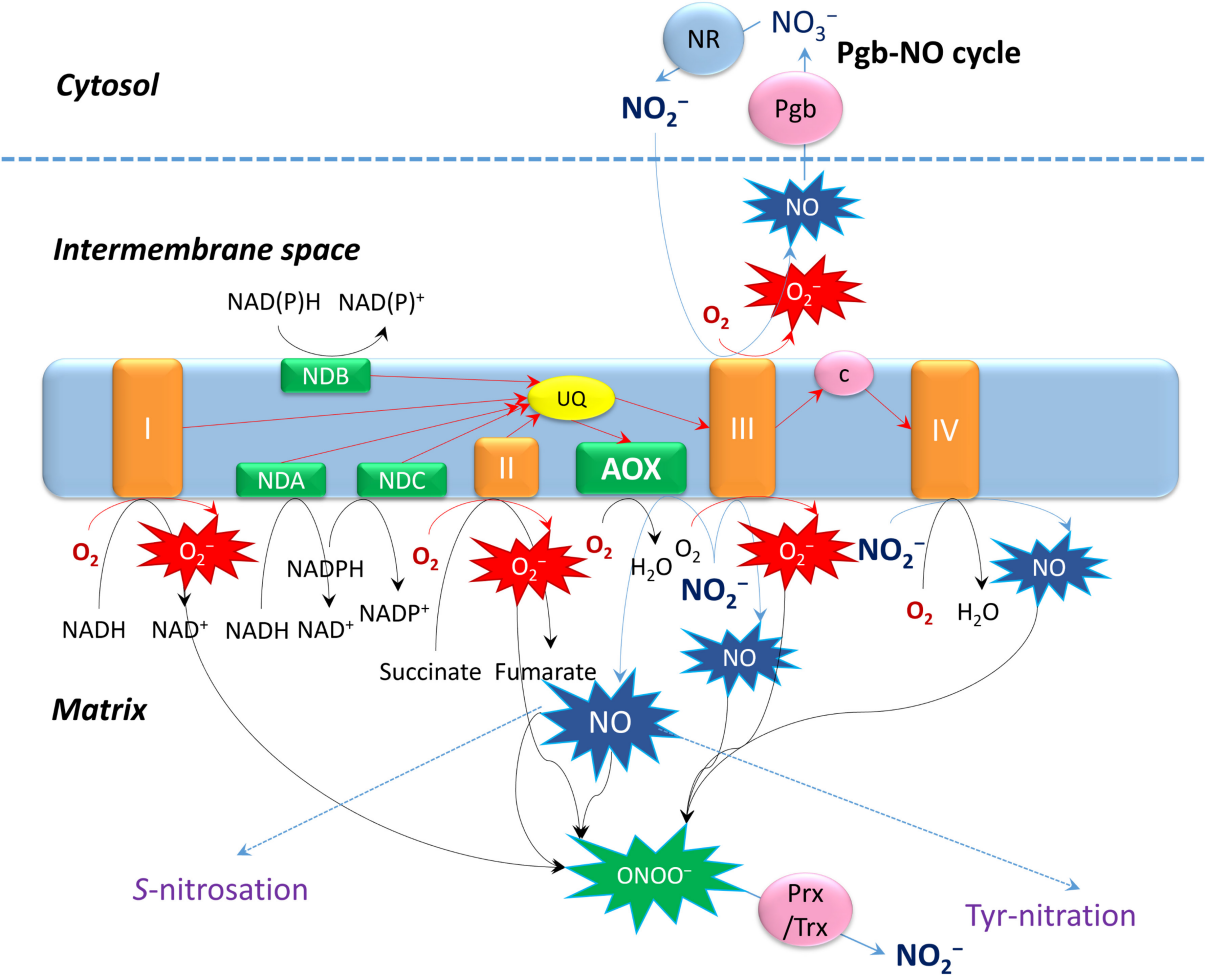
Operation of alternative oxidase in mitochondria under oxygen-limiting conditions. Abbreviations: I, II, II, IV, electron transport chain complexes; AOX, alternative oxidase; NDA, NDB, NDC, alternative NAD(P)H dehydrogenases; NR, nitrate reductase; Prx, peroxiredoxin; Trx, thioredoxin; c, cytochrome *c*; ONOO^−^, peroxynitrite; Pgb, phytoglobin.

The operation of AOX in mitochondria under oxygen-limiting conditions is presented in Fig. 4. While under normoxia, AOX prevents the formation of superoxide anion and NO by diverting electrons from the cytochrome *c* pathway (Complexes III and IV), and thus keeps the redox level in the cell under control (Del-Saz *et al.*, 2018; Igamberdiev and Bykova, 2023), under hypoxic conditions, AOX participates, together with Complexes III and IV, in the production of NO from nitrite. It contributes to the turnover of the Pgb–NO cycle and to the formation and degradation of peroxynitrite (ONOO^−^), and regulates the processes of *S*-nitrosation and Tyr nitration (Astier and Lindermayr, 2012). The formation of superoxide anion by Complexes I and III under oxygen-limiting conditions at the elevated redox level is balanced by its scavenging in the reaction with NO, followed by the peroxiredoxin/thioredoxin-dependent degradation of the formed peroxynitrite (Wulff *et al*, 2009). While operation of the Pgb–NO cycle and peroxynitrite scavenging result in the consumption of NAD(P)H, this function of AOX also facilitates the maintenance of redox balance. Inhibition of aconitase by NO (Gupta *et al.*, 2012) leads to the suppression of the tricarboxylic acid (TCA) cycle and citrate efflux from mitochondria, which activates the expression of AOX (Vanlerberghe and McIntosh, 1996) and results in further facilitation of the Pgb–NO cycle (not shown in Fig. 4). The subsequent stimulation of glycolysis via reoxidation of the glycolytic NADH in the Pgb–NO cycle diverts metabolism to the synthesis of amino acids and to the maintenance of major physiological processes under oxygen-limiting conditions.

## Conclusions

Even though AOX features a low affinity for oxygen, it remains an important player under hypoxic conditions. This non-energy-conserving terminal oxidase participates in NO turnover via the Pgb–NO cycle and is possibly involved in the production of NO from nitrite, similarly to other redox proteins. This process facilitates the oxidation of NAD(P)H and thus regulates redox balance in the hypoxic cell. Operation of AOX results in the avoidance of lactic and ethanolic production in the course of glycolytic metabolism by diverting pyruvate conversion to the formation of Ala. The latter is further transaminated, and the pools of different amino acids are formed. Metabolic processes controlled by AOX are important at different stages of plant development and in the course of plant adaptation, in particular during seed germination, in meristematic tissues experiencing oxygen depletion, under flooding conditions, and in other stress responses associated with oxygen deprivation.

## Abbreviations

AOX: alternative oxidase
COX: cytochrome *c* oxidase
ETC: electron transport chain
GABA: γ-aminobutyric acid
GDH: glutamate dehydrogenase
DAF-FM: 4-amino-5-methylamino-2’,7’-difluorofluorescein
NO: nitric oxide
Pgb: phytoglobin
